# Mr. William Robert Meyer

**Published:** 1910-06-25

**Authors:** 


Mr. William Robert Meyer, surgeon on board s.s. *Fulani,* has died at sea. He became an L.S.A. in 1894.

